# The Hospital. Nursing Section

**Published:** 1904-04-09

**Authors:** 


### The Hospital.

### Nursing Section.

Contributions for this Section of "The Hospital" should be addressed to the Editor, "The Hospital" Nursing Section, 28 & 29 Southampton Street, Strand, London, W.C.

No. 915.—Vol. XXXVI. *SATURDAY, APRIL* 9, 1904.

### Notes on News from the Nursing World.

### THE NEW LADY SUPERINTENDENT OF THE NURSES' CO-OPERATION.

We understand that Mrs. Lucas has been appointed lady superintendent of the Nurses' Co-operation in succession to Miss Roberts. Mrs. Lucas was, before her marriage, Miss Florence Pepper and occupied for some time the office of matron of the Royal Devon and Exeter Hospital. On her marriage to Lieut.-Colonel Lucas, the then president of the Board of Governors of the hospital, she retired, but, after the death of her husband, she resumed her nursing work and has for some little time been matron of the Coventry Hospital. She is a capable and experienced lady, and it is to be hoped that, as she was successful at Exeter, she will be able to master the numerous problems surrounding the difficult post she has accepted.

### JAPANESE NURSES TRAINED IN CANADA.

It is stated that Dr. Omari Kroe, of Chicago, has been commissioned by the Japanese Government to recruit nurses for the field hospitals. But they are to be limited to the Japanese themselves, and Dr. Kroe expects to obtain as many as he requires in Montreal, where Japanese women and men are trained as nurses. With respect to the persistent offers of American women to nurse Japanese sick and wounded, Dr. Kroe says that it is believed by the war department that long before Americans could become familiar with the Japanese language—which is a necessity—the war will be over.

### SALISBURY NURSES' HOME AND THE PENSION FUND.

An interesting feature of the annual meeting of the subscribers to the Salisbury Nurses' Home was that Lord Pembroke, who presided, prefaced his speech by reading a letter from Earl Nelson expressing his great satisfaction with the conduct of the nurse from the Home who nursed him during his recent illness. Lord Pembroke, who paid a high compliment to the lady superintendent, Miss Lawrence, and said she was emphatically the right person in the right place, dealt at length with various features of the report. Referring to the contribution of £150 to the Royal National Pension Fund for Nurses, he affirmed that "it was most desirable, and would add largely to the benefits of the Home." We do not doubt that this will be so, and that the wise policy of the committee in affiliating the Home to the Pension Fund will enable them to command the services of the best trained nurses.

### DISTRICT NURSING IN JERUSALEM.

We hear good accounts from Palestine of the Nurses' Home at Jerusalem, which was opened last May. The Home is in connection with St. George's College. At present the nursing staff consists of a lady superintendent and two certificated nurses. They attend district and private cases, and afford relief to poor Mahomedans, even visiting Mahomedan women in prison. The latter are particularly grateful patients; and by means of this new channel the practical value of Bishop Blyth's mission should be appreciably augmented.

### NURSES AND AGENCIES.

The position of an agency offering employment to nurses does not seem to be fully understood. We have lately received complaints from nurses that they have been sent by agencies to cases which were not of a genuine character. But it does not follow that the agent had any knowledge of the fact, for we find, as the result of many inquiries, that it is frequently the habit of agencies to send nurses who apply to them to the addresses of persons whose advertisements have appeared in various newspapers. Thus, we have before us a form forwarded to a correspondent by an agency in London, in which the only information given is that a lady, whose country address is given, wants a nurse to go abroad, and it is added "no further particulars known." This is very candid, but it shows quite clearly that a nurse consulting an agency with the view of obtaining employment must not take for granted that any responsibility whatever is accepted by the latter beyond that of trying to place the parties in communication with each other. After this nurses should make careful and strict inquiries themselves, and doubtful addresses and uneducated handwriting should be treated with suspicion.

### MATRON'S SIGNATURE AND THE LOCAL GOVERNMENT BOARD.

The refusal of the Croydon Guardians to reinstate the matron's signature on the probationers' certificates has again given rise to discussion. In one form or another the question is being continually raised. A few weeks ago the probationers now in training petitioned the Infirmary Committee, asking for the revision of the present form of certificate. The Committee recommend compliance with the request. The Board, by the casting-vote of the Chairman, declined to give way. At the last meeting of the Infirmary Committee the issue cropped up owing to the fact that Mabel Bere, a probationer on three months' trial, had left the infirmary, her father having found on inquiry that she would be unable to obtain a post in the Army Nursing Service with the form of certificate granted to her at Croydon. A letter was read from Captain Bere on the subject. The Committee recommended that the order of the Board, "that officers of the Board shall not give testimonials to their subordinate staff," shall not operate in the case of the matron of the infirmary, who shall be authorised to sign a supplemental certificate for nurses, if she desires to do so, stating that the nurse has served her three years to her satisfaction. It was decided by the Board that this recommendation of the Infirmary Committee should be specially dealt with *in camera* at the close of other business. Subsequently it was thought well to adjourn the discussion to a future meeting. As the Local Government Board recognise the Croydon Infirmary as a training school, it seems time that they intervened and settled the question once for all.

### RAISING THE STANDARD AT SIR PATRICK DUN'S HOSPITAL.

We are glad to hear that the Board of Governors of Sir Patrick Dun's Hospital, Dublin, have decided to increase the term of training for probationers from two to three years. In future, therefore, the certificate of this well-known institution, of which Miss L. V. Haughton is matron, will be accepted as a qualification for posts in Queen Alexandra's Imperial Military Nursing Service, and, in fact, render nurses who hold it eligible for any appointment at home or abroad.

### THIRTY YEARS' WORK AT GLASGOW.

It is 30 years since Miss Agnes M'Alpin started the Glasgow Training Home for Nurses, and at the annual meeting the chairman, in submitting the report, conveyed to Miss M'Alpin the congratulations of the subscribers on the fact that she is still actively engaged in the duties which she had made the work of her life. Since its opening in 1874 there have been registered in the books of the home 13,426 cases nursed in private families, and 7,241 cases nursed in the home. In 1903 the number of the former class of cases was 559, and of the latter 344. The home started with seven nurses; there are now 106, of whom 79 are fully qualified. A very large number of capable women have also been trained under Miss M'Alpin's care and certified as competent by the visiting medical officers since the home was founded. It has now become necessary to carry out a scheme of reconstruction, and we trust that at an early date Miss M'Alpin will have the satisfaction of seeing a new house large enough for present needs in existence.

### A PLETHORA OF NURSES AT BRISTOL.

The report of the annual meeting of the Bristol Nurses' Institute certainly suggests that the supply of nurses in that city is overdone. In the early summer of last year, the report states, there were as many as twelve nurses for weeks without cases; and again in the early winter there was very little demand for their services. That this was not due to any prejudice against the nurses of the Institute is shown by the fact that the experience of other institutions in Bristol was similar. Possibly the simple explanation is that fewer people required nurses, but it is also conceivable that the increasing difficulties of making two ends meet compelled persons who required to be attended by a nurse to dispense with one. The experience of the Bristol Institute, which does not stand alone, may therefore be a warning that private nursing has its limits.

### A WISE DECISION AT IPSWICH.

In his speech at the annual meeting of the Suffolk Nursing Association, held at Ipswich, the chairman said that the Committee had been pressed to allow the nurses' home, which was formally opened by Princess Christian last year, to be used for the purpose of operations on paying patients. The Committee have, however, declined to entertain the idea, and we think that they are absolutely right. The conditions on which the building was erected was that it should be carried on as a nurses' home, and the Committee would have been distinctly to blame if they had permitted it to be utilised for the purpose of conducting operations. We are rather surprised that such a proposal should in the circumstances have been put forward. It is satisfactory to learn that the work of the association is much appreciated and admirably done, but new subscribers are badly wanted in order to keep the financial position sound.

### A BRILLIANT SUCCESS AT NOTTINGHAM.

With a population of nearly a quarter of a million Nottingham possesses only 13 nurses engaged in district work. The result is that, despite the most energetic efforts on the part of the staff, no nursing can be done in some of the poorest and most crowded parts of the town. Recognising the urgent need for further assistance in alleviating the distress amongst the sick poor, Lady Maud Rolleston, whose work in South Africa during the war is still gratefully remembered, came to the rescue, and organised two concerts at which many of the leading London and provincial vocalists and instrumentalists appeared. The Duchess of Portland entertained the artists, who kindly gave their services, at Welbeck Abbey. The result of the concerts was that £343 has been handed over to the Nottingham and District Nursing Association towards the employment of additional nurses. It is estimated that a sum of £500 will provide for two additional nurses for three years, and by that time it is hoped that their services will have been so much appreciated that the necessary money will be raised and an increased staff maintained.

### A WELCOME GIFT.

Moretonhampstead, the little town at the edge of Dartmoor, has received a very welcome gift. In memory of his wife, Mr. George Wills, of Pepperdon, has not only erected, but fully furnished and equipped, a nurses' home. It occupies a prominent site in the town, and is well adapted for the purpose. The building was designed by the late Mr. Silvanus Trevail, and it will be occupied at once.

### GRUDGING THE NURSES A REASONABLE HOLIDAY.

At a recent meeting of the Bristol Board of Guardians, Mr. F. Weaver moved that three weeks' leave of absence be allowed annually to the nurses in the workhouse hospitals. Last year, for the first time, a holiday of three weeks was conceded. He thought that nurses who had to work about 80 hours per week needed a holiday of three weeks, and it had already proved beneficial to their health. Mr. Burland proposed as an alternative that three weeks' holiday be granted this year, and the amendment was accepted. But Mr. Gale desired to adjourn the question in order to make inquiries as to holid ays in other large unions. Canon Griffiths remarked, not without cause, that the opposition was astonishing, and expressed the hope that the guardians would grant three weeks' holiday to girls who richly deserved it. As only ten supported the adjournment against thirty-five in favour of the amended motion, the nurses will have their full holiday this year. But there seems to be no reason whatever why the arrangement should not be made permanent.

### "A DISGRACE TO CAPETOWN."

The Governor of Cape Colony presided at the annual meeting of the Victoria Nurses' Institute, which was held at Capetown a few weeks ago, and himself read the report. Although His Excellency spoke of the satisfactory state of affairs revealed by the report, Dr. Jane Waterston did not by any means adopt an optimistic tone, but dwelt in strong terms upon the necessity of at once rebuilding the old wing of the institute, in which, by the collapse of an outer wall, 13 apartments have been rendered untenable. She declared that it was "a disgrace to Capetown that the most valuable nursing institution in the Colony should show a broken wall for so long," and she insisted that the money ought to be found at once to build it up. We trust that the Governor's panegyric on the work done by the nurses may have the effect of prompting the people of Captown to remove the reproach which at present attaches to them.

### COUNTY ASSOCIATIONS AND FULLY-QUALIFIED NURSES.

There is a danger lest the county nursing organisations which have lately been formed, in their anxiety to provide the sick poor with nurses, should under-estimate the importance of adequate training. It is therefore satisfactory to note that the chairman of the Somerset County Nursing Association, which has just held its second annual meeting, said it was not the object of the managers to introduce partially-trained nurses, but, where possible, to induce the affiliated associations to employ fully-qualified nurses. Of course, we know that the cost of employing a fully qualified nurse is greater, but the labourer is worthy of her hire. Moreover, the difference of the annual cost, which in the one case is from £80 to £100 and in the other from £45 to £60, though substantial, can generally be made up by subscriptions. The authorities who can assert that they have secured the best nursing talent which can be obtained, naturally appeal for contributions with much more force than those who have to plead that they can only afford to pay the salary of an imperfectly-trained nurse.

### THE COUNTESS OF DUFFERIN'S FUND.

In the annual report of the United Kingdom branch of the Countess of Dufferin's Fund it is mentioned that the past year has seen the successful establishment of the Victoria Memorial Scholarship Fund for training Indian midwives, for which about 650,000 rupees has been raised by Lady Curzon. But the main feature of the year is "the marked improvement which is reported in the financial condition of the association, and especially the progress made in Bombay." We are glad that this excellent organisation, to which the Dowager Marchioness of Dufferin and Ava continues to devote her constant attention, is obtaining the support it so thoroughly deserves.

### EXAMINATION OF PROBATIONERS.

At the recent final examination of probationers who have completed three years' training at Islington Infirmary, Highgate Hill, 19 candidates succeeded in satisfying the examiner. The examination was conducted by Mr. Raymond Johnson, F.R.C.S., Surgeon to University College Hospital, and a very satisfactory report was made by him. Apart from the result of the final examination, bronze medals are awarded to probationers at the end of the first year's training provided that the general conduct has been good.

### A DEFICIENCY AT WEDNESBURY.

For the first time during the six years which the Wednesbury District Nursing Institute has been in existence, the Committee had to announce at the annual meeting that the balance of expenditure for 1903 was in excess of income to the extent of £47. This, if it stood alone, would be a very serious deficiency, seeing that it represents an eighth of the annual receipts, and that the expenditure for the year was only normal. But it appears that the sum of £33 15s., due on account of some sports, had not been paid over at the end of the year. The deficiency, therefore, is small. Still, it is a matter for regret that there should be any indication of retrogression, and we think that the persons responsible for the sports should have taken care to send in their cheque before the end of the year. The working classes cannot be accused of not giving adequate support to the movement, for they contributed the respectable sum of £140, or nearly £20 more than in 1902.

### A LEGAL POINT.

At the last meeting of the Buckingham Guardians a letter from the Buckingham Nursing Association asking that one of their nurses, who is a certified midwife, might be allowed at once to obtain the services of the district poor-law medical officer in difficult cases, and afterwards secure the necessary order, was considered. The clerk to the Guardians having stated that they could not legally acquiesce in the application, it was thereupon refused.

### SHORT ITEMS.

In consequence of ill-health Miss Helena A. Gomme has been compelled to resign the post of Lady Superintendent of the Prudhoe Memorial Convalescent Home, Whitley, Newcastle-on-Tyne.—Miss W. M. Jay, Miss F. M. MacGregor, and Miss M. MacGregor, have been provisionally appointed staff nurses in Queen Alexandra's Imperial Military Nursing Service.—At a recent examination held at the Clayton Hospital, Wakefield, by Mr. J. W. Walker, the following nurses passed with distinction: Nurses Lillie Reynolds, Dorothy Richardson, Florence Parkinson, and Annie Ward.

### The Nursing Outlook.

"From magnanimity, all fear above;
From nobler recompense, above applause,
Which owes to man's short outlook all its charm."

### NURSING IN PARIS.

It must always be remembered with regard to strictures on, or articles in praise of, the hospitals of Paris, either as regards nursing, medical treatment, or administration, that these institutions are municipal, and therefore apt to be used as pellets in political warfare. It is one of the strongest arguments against municipalisation of hospitals that where this has taken place these institutions have been harassed by attacks that were due solely to a desire to secure votes or to damage certain councillors. This word of warning seems necessary because some of us who have worked in, or been nursed in the hospitals of France, have been much astonished at the unfavourable articles that have found their way of late into foreign and English and American journals. Here we have only to deal with the nursing side, and its difficulties are such as we can well appreciate.

First, let it be understood that hospital nursing as an outlet for a cultivated woman's desire to do her share of the world's work, does not exist in France; the enthusiasm for humanity when it awakes in the heart of some young Parisienne takes her as a rule into a convent, and the religious sister has been ousted from the hospitals of Paris. This is the root of the whole matter—the religious orders are not popular with one political section in France, and the nuns were removed during the end of the last century and replaced by lay nurses. But the lay nurses had to be drawn largely from the uneducated class, and for a long time the directors of the Assistance Publique, which manages all the hospitals, had hard work to get suitable women. Even now they cannot get the cultivated and educated women to enter their wards whom they would like, though they are making a strong effort to secure a few lay sisters of higher standing. They are, in fact, doing just what we had to do in London at one time, when the "lady nurses" came on duty late, wore a distinctive dress, only trained for one year, and were generally promoted rapidly. It is in vain that Dr. Bourneville and others have written nursing text-books, started lectures, tried their best to train and ennoble the bourgeoise nurse; she, good woman, has made a useful substitute for the nuns, she is obedient to the doctors, cleanly and economical, kind to her child patients, cheerful with all—but as for scientific knowledge and efficient training—no indeed! Nursing is not the fashion in France, and no man, be he doctor or no, can alter the fashion for women. So with a sigh the Assistance Publique has determined if it cannot educate the rank and file to the level of the English or American nurse, to at least try and get a few superior and intelligent women to take the higher posts. They are therefore building a handsome "college of nursing" in connection with the Salpêtrière Hospital; and here the probationers will have good bedrooms, sitting-rooms, library, lecture-room, and all the housing and teaching advantages of an English "nurses' home." Their practical teaching will not be so good, as they will go daily, after preliminary training in the wards of the Salpêtrière, to other hospitals for surgical, fever, and further experience. Still, on the whole, the move seems to us a good one: the better housing arrangements will attract more refined women, and once the cultivated classes take to nursing as a profession the main object will be accomplished. Meanwhile, it scarcely seems fair to censure Dr. Bourneville, M. Henri Monod, and others in power: some of the condemnation should fall on the women of France, who have not yet seen their duty towards the sick poor, and seem to give all their sympathy to poodle dogs.

Without proper material it is impossible to produce satisfactory goods, and after all we have seen some excellent nursing in the Enfants Malades and in the Maternité of the St. Antoine, to speak from personal experience only. Nothing could exceed the excellent and up-to-date arrangements at the Maternité; the fittings are all glass and cleaned to perfection, and the work of the midwives is in many ways superior to that of the English midwife.

It is so easy to criticise; it is so natural to think our own ways and our own country right, and all other methods and all other conventions wrong. But our own highly-trained and taught nurses might learn something from the French nurse, in her cheerful readiness to do all that comes to hand, and to do it with economy and with kindness.

Personal influence counts for much; it is far easier to get up enthusiasm for a woman than for a cause, and in England the graceful heroic figure of Florence Nightingale did in two years what the Assistance Publique has not been able to do in twenty. Perhaps, had there been some lady of position in Paris to interest and advise, to suggest a becoming uniform for the hospital nurses, to arrange receptions and social homage for them, things might be moved more quickly. Seeing how little the Paris nurse has beyond her work and her salary, it is hard to demand that she should be a scientific saint: she is generally a good hard-working woman, and if the new college develops a higher class of nurse, we hope that in the future the steady kindliness of those who have done their best during the trying reorganisation of nursing in France will not be forgotten.

### Lectures Upon the Nursing of Mental Diseases.

By Robert Jones, M.D.Lond., B.S., F.R.C.S.Eng., M.R.C.P.Lond., Resident Physician and Superintendent of the London County Asylum, Claybury.

### LECTURE IX.

In the preceding lectures we have dealt with the normal functions of the body, but our attention will be devoted now to variations in the healthy performance of these functions and their treatment, as also to the relation which the organism—*i e.* the individual, the coherent whole, or the sum-total of these functions—bears to the surroundings, or, as these have been called, the "environment."

The attempt to convey a notion of insanity to a person who has never experienced or seen any cases of mental unsoundness is not an easy task, and this is the more difficult insomuch as there is no definite standard of sanity fixed either by Nature or by any recognised authority, and there is no line of demarcation between sanity and insanity. The phenomena expressed by health on the one side and disease on the other are indefinable. Health and disease are ultimate facts of human experience, and imply something indefinite, like life, and to call disease a "perverted life process" is no assistance. If health may be described as the easy and harmonious performance of all the natural functions of the body or an absence of those diseases, over a thousand in number, which are entered in the nomenclature list of the Royal College of Physicians, then disease may be defined as a "negation of health." As in bodily health there is no person who is absolutely well, so mentally there is no one perfectly sane in all his mental faculties. Among our acquaintances there are some persons who, although exceedingly able and clever, with ability amounting even to genius, are yet in some ways eccentric or peculiar. They are erratic, and behave in ways that are not those of the ordinary individual. There are wide departures from a fixed standard in the life of every individual, and the mental state of a person, indeed of every person, varies from time to time under different conditions or circumstances. In relation to his enemies the mental state of an individual may be a combative one, in relation to superiors it may be receptive or submissive, whereas in relation to inferiors it may become a controlling and dominant one. The mental state in childhood, again, is different from that in youth; youth is different from maturity; and this, again, from the mental state in old age. We shall later on see the significance of these statements. These variations are not regarded as unhealthy, or as symptoms of insanity, so long as they do not pass beyond certain limits. Further, social circumstances, education, and culture leave their impression upon man's mind, so that in the investigation of insanity it becomes necessary to consider the person's *status* in society, his age, the time and place in which he lives, and his previous conduct and position. Conversation and conduct which would be consonant with sanity in a "navvy" or a huckster would be strong indications of insanity in an educated and cultured person, or in a man of affairs. Beliefs which in the earlier periods of history would be the natural creeds of the majority would to-day probably be taken as insane delusions. The behaviour of youth, with its romance and exuberance of phantasy, its poetry and its extreme light-heartedness, would in an old person possibly suggest the onset of incipient insanity. Conduct which would be proper and suitable in one place or upon certain occasions would under other circumstances be a symptom of insanity—for example, the language and levity of a music-hall performer would be viewed as mental unsoundness if seen in the conduct of an episcopal dignitary. The portly tradesman who, instead of serving his customers, began to play leap-frog over the counter would be looked upon as mad. The possessor of riches or property who denied himself the necessities of life, or who outraged decency by casting off his clothes in the public streets, would also be an instance of an affection of conduct showing a lack of reasoning power and of a proper understanding of the fitness of things. Conversation and conduct are therefore the keys to the interpretation of the mind, and it is from these that we obtain the indication of insanity. There are certain factors in the individual which help us to realise this want of adaptation to surroundings, and these are, more especially, the expression and the general appearance of our patient, who may be bright and lively, or dull, depressed, and listless. The expression and the posture give us much indication as to the mental state; and especially is this noticed when questions are put to him and the mental reaction is observed. It is then observed whether he understands what is said to him, and whether he answers intelligently and correctly. It is mainly upon the above data that we judge whether a person is sane or insane, and whether he may be suffering from delusions which control his actions and alter his normal habits. Delusions by themselves are not an actual sign of insanity, although they may be a symptom. There are many noted persons—Joan of Arc, Peter the Great, and Warren Hastings, for instance—who have had delusions which they cherished and in which they believed, yet they were able to carry out—and with distinction—all or most of their particular duties in life.

In the growth and development of the individual certain instincts are implanted in man's nature which are characteristic of his higher development, and these have two aspects as either relating to himself or to his surroundings. The first set of instincts relate to his self-preservation—*i.e.* to his own support and to his search for food, warmth, and shelter; the second relate to his reproduction and to the care of his offspring. These are, again, subordinated to the welfare of the community, and have a bearing upon his surroundings. A community, or men and women living together in association, have certain conventions, ceremonies, and courtesies which have to be observed, and which are thus indirectly self-preservative to the individual. Society makes certain rules for its own protection, to which all (and therefore each individual) are expected to conform; but when conduct deviates from the standard fixed for it by society, and there is interference either with his fellow-creatures, their property, or their comfort, then the question of law-breaking or insanity, of which there are many varieties, has to be considered.

It is convenient for the nurse to have some knowledge of these different kinds of insanity. One form of classification is according to the cause, such as epilepsy, alcohol, and the puerperal period. Such a classification may not only cause confusion—for it is not invariably possible to say that insanity is always the result of one cause—but also it may react upon the patient's infirmity, and, by directing special attention to the cause, may aggravate the symptoms. A reference to the dangers of the puerperal period may excite morbid fears during some part of every subsequent pregnancy, and may not only affect the parent but may also react upon the offspring. Again, attention called to fits may bring them on, as worry, anxiety, and fear are known to do. A more convenient form of classification is according to the age of the patient, such as the mental unsoundness which occurs during (1) the developmental period of childhood—and this carries with it some suggestion of the forms met with, such as imbecility, idiocy, or the effects of epilepsy, and the prognosis in each of these is bad; (2) the insanity of growth which occurs during the period of adolescence, that of (3) maturity (4) the climacteric crisis of life, in which all the abnormal mental forms are met with, marked either by excitement, depression, or mental impairment, including that special form termed general paralysis of the insane, which occurs at the period of greatest vitality, and to which we shall refer later. Lastly (5) the insanity of senile decay, which accompanies the onset—and may be only an exaggeration—of ordinary and natural old age.

Some have classified insanity as curable and incurable; others have referred to it as acute or chronic. Some have referred to varieties of insanity which have been merely the reversal of mental faculties, such as moral perversion, suicidal insanity, will-less or impulsive insanity. Whatever system is adopted, there will probably be some cross-classification, and some symptoms are bound to be in each class. The great point s to abridge distinctions, with a view to establishing well-defined groups having a maximum significance; and of all classifications, that based upon the symptoms is the best, and the one we shall adopt, viz.: (1) States of depression or melancholia, which include simple and chronic cases and those of fixed delusions with depression. (2) States of excitement or exaltation which involve acute and chronic mania, and cases of fixed delusions which present exaltation. (3) States of mental weakness, such as idiocy and imbecility in children, and the primary, secondary, or terminal dementias of young persons and of adults. (4) States of stupor. (5) States of impaired will-power, which include uncontrollable, sudden, and violent impulses, together with cases exhibiting what are called "fixed ideas." (6) Epilepsy, with its subsequent loss of mind. (7) General paralysis of the insane, ending in complete dementia, bodily decay, and death.

The above is a useful and suggestive classification. It will give the nurse some idea as to the treatment required, and will be more helpful as involving less confusion through the use of technical terms. The general duties of the nurse upon the insane will be dealt with in the next lecture.

### Opening of a Nurses' Home at Lambeth Infirmary.

The formal opening of a new Nurses' Home which the Lambeth Guardians have erected at Brook Street, Kennington, for the nurses of Lambeth Workhouse Infirmary, took place last week.

The ground floor of the building—which is of red brick faced with white stone—is mainly occupied by the offices of the Guardians themselves, so that the entrance to the Home is not of an imposing character. This, however, is a matter of no moment, as the nurses, except when they have been out, naturally use the covered way which connects the Home with the Infirmary. The covered way will be greatly appreciated. Hitherto, many of the nurses have perforce been accommodated across the street in two houses rented for the purpose, and the constant crossing of the quadrangle and the road in all weathers has, of course, been attended with much discomfort. As yet there are several little details of the Home not quite finished, but it was imperative that a move should be made at once, as the lease of the houses in which the nurses slept ran out at Lady Day. Accordingly, 50 of them are already in their fresh quarters, the others remaining in the Infirmary until the workpeople are finally ousted.

The space on the ground floor is somewhat limited. There is a small courtyard which at present is chaos, but will shortly be laid down with grass and made to look attractive. The matron, Miss E. M. Byles, is very anxious, if possible, to get enough space for a croquet lawn for the nurses, but as a carriage drive for luggage, etc., is absolutely essential, she is afraid that her project may have to be abandoned. A bicycle shed stands in the corner of the square. The dining-room, kitchen, mess-room, pantry, visitors' room, and cloak-room are all downstairs, but, with the exception of these five rooms, the rest of the Home is above.

The dining-room is of an oblong shape, with walls of a pale *café-au-lait* hue, while the paint is white. Sixty nurses can dine at a time, and serving should be an easy matter, as the kitchen is next door. Here are three gas ovens, as well as a large open range, which, it is calculated, will suffice comfortably to cook all the meals for the nursing staff, their culinary arrangements being entirely separate from those of the infirmary. The matron's quarters will, however, still remain where they are, the assistant-matron and home-sister being located at the Home. The mess-room pantry—where the glass and silver is washed up—has one great convenience: it is a steamer in which six gallons of water can be made to boil in four minutes, and is specially placed there so that the nurses can make themselves tea when they require without giving any trouble to anyone and without the risk of using that dangerous little utensil, a methylated spirit stove, in their bedrooms. The visitors' room is only a small one, but should be welcomed by those who want a quiet chat, and, situated as it is just inside the door, should prove handy. The cloak-room, an excellent idea, is fitted with eight lavatory basins with hot and cold water, so that nurses can wash their hands *en route*, as it were, from the infirmary to the dining-room, and have no occasion to go up to their bedrooms. Mention may be made of another convenience. Long stands, somewhat similar to those with which a mantle-room in a big shop is fitted, run all down one side, each arm possessing six or seven big hooks. Here all damp cloaks or wet skirts can be left to dry, keeping the bedrooms both drier and cleaner in consequence.

The first floor is reached by a stone staircase, which looks clean and cool with its deep cream shiny tiles arranged as a dado, then a warm brown wooden rail, the wall itself being temporarily painted green. After twelve months their colour may be changed, or renewed permanently. The floor of the passages, as well as that of the sitting-rooms and bedrooms, are all of polished wood. The recreation room is large and light, airy, and yet, with its lounges and small tables and its two huge fireplaces, as well as radiators to warm it in winter, very cosy withal. The sisters' sitting-room occupies the head of the T in which the Home is built, and is furnished almost entirely with arm chairs, either of the tall, roomy shape known as "Grandfather" or of an easy wicker kind. The dark peacock-green chintz contrasts pleasantly with the cream walls, this being the prevailing hue in all the sitting-rooms. There is also a small sitting-room for the maids, a most business-looking lecture-room, and bedrooms for 20 nurses. On the second floor are bedrooms for 16 sisters and 24 nurses, whilst the top floor takes 40 more nurses and the maids' apartments. The night nurses are accommodated at the top of the house, and their rooms are quite shut off from the rest of the house. All the bedrooms are similar in colouring, the walls being French grey and the paint white. They vary in size according to locality, and the sisters' rooms are distinguished by containing an easy chair in addition to the other furniture. The latter includes a brass bedstead covered with a white counterpane having a red band top and bottom, which looks nice against the grey walls and harmonises with the fitted toilet covers of red American cloth. The latter are used instead of white dimity for the dressing-table and chest of drawers to save the continual washing. For the same reason muslin blinds are dispensed with, the bottom pane of glass being thick, so as to render it impossible for the occupant of the room to be overlooked. Each bedroom—and everybody in the Home has a separate one—is also fitted with a wardrobe, a marble-topped washing-stand, a three-cornered basket for soiled linen, and a square of red carpet. No nails are allowed in the walls, but a picture rail is provided. There are three bath-rooms on each floor and a lavatory basin, also a box-room, and a telephone which communicates everywhere—to the infirmary, the mess-room, the kitchen, the matron's room, etc. There are appliances for extinguishing fire on each floor, and the nurses are drilled by an efficient inspector each month; there are three inside stone staircases and three outside iron ones for emergency exits, and last, but not least in the estimation of the nurses, one flat roof on the first floor and two on the second. These flat roofs are to be furnished with garden chairs, little tables, boxes for planting flowers, which the nurses will probably enjoy making gay and bright. And then, when everything looks smart and inviting, perhaps the warm days of summer will really come, and it may be safe to prophesy that "roof tea-parties" will become the fashion amongst the nurses of the Lambeth Infirmary.

### Appointments.

[No charge is made for announcements under this head, and we are always glad to receive, and publish, appointments. The information, to insure accuracy, should be sent from the nurses themselves, and we cannot undertake to correct official announcements which may happen to be inaccurate. It is essential that in all cases the school of training should be given.]

Fulham Infirmary, Hammersmith, W.—Miss Frances C. Wallen has been appointed home sister. She was trained at the Metropolitan Hospital, London, and has since held the posts of ward sister, and for the last two years night superintendent at the Southwark Infirmary, East Dulwich. She holds the L.O.S. certificate.

Liverpool Sanatorium, Frodsham.—Miss E. Wilson Ross has been appointed sister. She was trained at the Royal Devon and Exeter Hospital, Exeter, where she was afterwards staff nurse and theatre sister. She has since been sister of female wards and theatre at Bethnal Green Infirmary, and assistant matron at the Leytonstone Schools.

Medical and Surgical Nursing Home and Institute of Trained Nurses, Manchester. — Miss Alice Ann Tinker has been appointed matron. She was trained at King's College Hospital, London, and has since been engaged in private and district nursing. Lately she has been engaged at a surgical home in Cheshire.

Prudhoe Memorial Convalescent Home, Whitley, Newcastle-on-Tyne.—Miss Henrietta Preston has been appointed lady superintendent and Miss Jane Phelps assistant matron. Miss Preston was trained at the Hospital for Women, Liverpool, and the General Infirmary, Stockport. She has since been charge nurse at the General Hospital, Stroud; sister at Parkwood Convalescent Home, Swanley; night superintendent at St. Mark's Hospital, London, and assistant matron at Prudhoe Memorial Convalescent Home. Miss Phelps was trained at the Clayton Hospital, Wakefield, where she was afterwards sister. She has since done private nursing in connection with Queen Victoria's Nursing Institution, Wolverhampton, and been night superintendent at the Clayton Hospital, Wakefield.

### Everybody's Opinion.

[Correspondence on all subjects is invited, but we cannot in any way be responsible for the opinions expressed by our correspondents. No communication can be entertained if the name and address of the correspondent are not given as a guarantee of good faith, but not necessarily for publication. All correspondents should write on one side of the paper only.]

### THE MIDWIVES ACT.

"E. M. B." writes: "Gante" does not seem to understand that a woman has to give references as to good character before she can be admitted for examination for the London Obstetrical Society certificate, or any other which qualifies her for registration as a midwife.

### PROGRESS AT BRISTOL.

"The Lady Superintendent of the Bristol District Nurses' Society" writes: With reference to your note headed "District Nursing as an Investment," it is true that this society began the year 1903 with a deficit, but we ended it with a balance at the bank of £17 0s. 5d.

[We are glad to make the correction. — Ed. The Hospital.]

### WANTED, A DISINFECTANT.

"Martha" writes: I am a subscriber to your paper, but I am not a trained nurse. I visit a great deal amongst the poor, and find the consequences most unpleasant in some cases. Could you recommend any precautions? I use a well-known powder freely, but it shakes off so quickly that it is not very useful. Is there anything else which would remain longer? I wear a short skirt; but even so, in these poor districts, I cannot say that it is a success.

### PORTRAITS AS PRESENTATIONS.

"E. H." writes: May I say that the idea of portraits as presentations seems to me to be capable of most interesting developments? Your correspondent "Delta" expresses the feeling of a great many nurses in her statement that replicas of presentation portraits of matrons and sisters in a board room or nurses' recreation room would act as a stimulant to nurses to deserve a similar honour. But why not replicas also of esteemed chairmen and governors, whose popularity with the staff would render both the presentation of a portrait and the production of replicas most acceptable? I am sure that the more this subject is discussed the greater will be the interest.

### PROTECTING MATTRESSES.

"A Sister" writes: Personally I have never seen mattresses used unless covered by a calico cover, which can be removed for washing. In the hospital where I received my first training all the mattresses were protected as follows: A white calico cover over mattress, a long mackintosh and sheet, across the middle of which was placed a small mackintosh and draw-sheet—in this way the mattresses were kept in excellent condition. At present I have under my charge 170 mattresses, all of which are covered by removable calico slips.

"Superintendent Nurse" writes: For preserving bedding and for keeping it clean I recommend removable slips, not only for mattresses but also for pillows, under the usual covers. For the former I use strong unbleached calico, and for the latter a soft thin kind, about 3d. a yard.

### STATE REGISTRATION.

Miss M. Preston, Matron, General Infirmary, Stafford, writes: If it is not too late I should like my name to be added to the manifesto against the State registration of trained nurses.

"Miss Amy Bullock, matron of the London Open-air Sanatorium," "Pinewood," Ninemile Ride, Wokingham, writes: As you are publishing names in The Hospital against the State registration of Nurses I should much like mine to be added, for I am quite sure that State registration would do far more harm than it would do good. It is a very easy matter for a nurse to conduct herself properly during her three years' training, but it is at the expiration of that period that nurses become indifferent.

Miss M. J. Dawson, Secretary of the Trained Nurses' Institution, Hyde Terrace, Leeds, writes: I shall be much obliged to you if you will inform me where I can obtain the memorial against the State registration of nurses, as I wish to sign it. I feel very strongly that it would be against the best interest of nursing and nurses also for such a Bill to become law; therefore I wish my signature appended to give strength to this protest. Several years ago we subscribed to the fund for litigation on this subject. I was pleased to observe on your list so many important names appended to this memorial.

### FILLING A WATER-BED.

"M. G." writes: I should like to ask some of your readers for their opinions and practice in the matter of filling a water-bed and placing a patient upon it. The matron of a surgical hospital discussed this question lately with me *apropos* of a recent occurrence in one of her wards. A patient suffering from serious spinal injury was ordered a water-bed, whereupon three of the sisters, trained in different hospitals, all of them well known and of good repute, were found to have the same idea on the subject, viz. that the empty bed should be drawn under the patient in the manner of a sheet, and then filled while the patient was lying upon it. This, they said, they had been separately trained to do in their schools. Considering how heavy and cumbrous a water-bed is this seems to me a most difficult and disturbing operation, whatever the nature of the case, but in some cases an absolutely dangerous one. The matron's training and practice had been the same as my own, viz., to fill the bed on an adjoining bedstead and then lift the patient carefully upon it. I was trained in the latter 'eighties—my friend, I believe, somewhat earlier—and we should both like to know if nurses are generally trained differently in this respect to-day. My opinion, frequently confirmed, is that although nurses may learn more now, they are not taught the essentials of nursing more thoroughly.

### ASYLUM NURSES.

"Mental Nurse" writes: I think that mental nurses generally will thank "An Asylum Matron" and also "A Mental Nurse" for their sensible letters in your paper lately. Mental nurses really are very slightingly spoken of by hospital nurses. We do not see why it should be so. Until recently mental nurses have had to work very much in the dark. It is not so very long since technical instruction has been given in the asylums, and in some places they still receive no tuition, though I think they should get the highest teaching, considering that they have to deal with diseases of the mind and nervous system. I am sure that they should also possess the highest principles. I am now in a large poor-law institution, and, as far as I can judge, the hospital and mental staff are much of the same type—working women earning their living in the most useful way they can, and their education and social standard much of a muchness. The Local Government Board forbid that an inmate shall attend on an insane patient; therefore, unless the staff is adequate, the work is hard and trying. The strain is great, for the hours are long, and in many cases the nurses are compelled to sleep surrounded by the patients at night, so that there is a feeling that one is never away from them. Some one must do the work, but why make it so hard and rough that the right sort of men and women cannot put up with it? There is no doubt that if the medical men in authority in this special branch would see that their nurses were treated on the same liberal lines as hospital nurses there would be less difficulty in securing high-minded women for the work.

### MR. JOHN LANGTON ON THE NURSING VOCATION.

"Fuzzy-Wuzzy" writes: In reference to a note in last week's *Nursing Section*, may I quote one paragraph from Mr. John Langton's speech? "He confessed that he should like to see even Jubilee nurses trained in hospitals for three years." Now, I think that this remark is misleading to the general public, as a nurse must have hospital training, frequently midwifery training, and, in addition, must give six months' probation in district nursing, sometimes longer before she is recognised as a *bonâ-fide* "Jubilee" nurse. Moreover, she has occasionally to wait for about a year before receiving her badge and brazzard from the Council. I may add that, as far as I can gather, the majority of the "Jubilee" or "Queen's" nurses are all nurses who have completed three and four years' training. Many of them have held staff appointments in their own training school or other hospital, and have eventually adopted district nursing as a distinct branch of their profession, because they have preferred it to taking further hospital appointments, or joining private nursing institutions, etc. I think that it is a good thing for district nurses to belong to the "Jubilee" or "Queen's" nurses, as it defines their position in the nursing world, and is a guarantee to the public that the nurse is properly trained, and that her work will be satisfactorily performed, as superintendents visit and inspect the different districts at regular intervals. It saves the nurse a great deal of trouble, as her interests are looked after by the Council, and she is left free to attend her district and duties, knowing that her interests are in good hands, and everything that is right for her will be done.

[The fact remains that all Jubilee nurses are not trained in hospital for three years.—Ed. The Hospital.]

### SIR WILLIAM BENNETT ON PRIVATE NURSING.

"A Matron" writes: In reply to "A Dublin Nurse" I am sorry to have to say that by her letters she shows a lamentable lack of knowledge with regard to the value of training. If she has been trained at all and knows anything about private nursing institutions she should be aware that as the applications for nurses are made to the matron—who is to a great extent responsible for her nurses—this is a matter which concerns matrons as well as nurses. She should also know that as the matron generally learns something of the nature of the case for which the nurse is required, she would, in any doubtful case if she were fully trained, be better able to thoroughly understand it and come to a fair decision as to whether it would be right to send the nurse, or withhold her, than she would be otherwise. "A Dublin Nurse" should also know that if after a nurse arrives at a case she has reason to feel that it is really unsuitable for a female nurse, although her matron is not at her elbow, she could and should communicate with her to that effect, either personally or by letter, when the matron, if she is fully trained, will be able to look at the matter from both a professional and a womanly standpoint and relieve the nurse of her doubts, either by withdrawing her from the case or showing her the necessity of remaining, as the case may be. If "A Dublin Nurse" cannot now see where the difference comes in I am afraid she is hopeless. But I should be sorry to think that there were many nurses who are unable to look upon the nursing of male patients from a higher standpoint than she evidently does. If that were so, the position of the poor male patients would be pitiable indeed. I think it would be a good thing—both for their own sakes and the sakes of male patients generally—if "A Dublin Nurse," and those who share her views, would, when their training is finished, either take up private nursing on their own account—when they would be at liberty to refuse to nurse any case they did not like—or seek posts in some of the special hospitals, for they would certainly be out of place in a general hospital or on a private nursing staff, except as obstetric nurses. Meanwhile, it is a pity that they should, by their unpleasantly suggestive remarks and wholesale denunciations, so misrepresent a large body of nurses, make the more delicate part of a private nurse's work more difficult, and also make it more difficult for male patients to meet their nurses with that absolute confidence which in many cases is such a great aid to recovery. It is for that reason, and in order to prevent a false and unpleasant impression being made on the minds of many highly respectable but sensitive male patients, and to show a nurse's work from a higher and nobler standpoint, that I felt it necessary to reply to the letters of "A Frequent Contributor" and "A Dublin Nurse." A good nurse should always be able to give a kind and intelligent response to the call of duty.

[This correspondence must now cease but the pamphlet to which "A Matron" refers is published by the Scientific Press, Ltd., 28 and 29 Southampton Street, Strand, W.C., price 6d., post free, 7d.—Ed. The Hospital.]

### THE LONDON HOSPITAL PRIVATE NURSING INSTITUTION.

The Hon. Sydney Holland, Chairman of the London Hospital, writes: In your leader last week you remark that the £4,211 paid to the London Hospital for its private nursing institution "presumably represents the profit made by the hospital from the earnings of the nurses." This is not accurate because as our nurses, between their cases, live with the other nurses and work in the wards, and as we have no separate nursing institution, we keep no separate account of the outgoings on their behalf in respect of board, lodging and service. It is therefore obvious that the figures you quote cannot fairly be taken to represent the "profit" which the London Hospital derives from its private nurses. You then add that "it cannot be entirely just to the nurses who earn the money, unless the London Hospital provides under a definite scheme which inalienably secures to the nurses a liberal provision in the day of sickness, and a liberal pension—say at 50 years of age." As a matter of fact the London Hospital does both. It gives its private nurses a pension of £55 per annum at 45 years of age, after 18 years' service, and this without deducting any portion of their pay to secure this pension. No better terms of pay or pension are given to any nurses by any hospital in England. Personally, I do not know of any as good. I wish I did. Our private nurses are paid £30, £35, £40, and £45 per annum with washing and everything found, including about £4 for unform. After six years from the date of entering the London Hospital as a probationer every member of its nursing staff receives an additional £5 per annum, which soon brings a private nurse's salary up to £50. After twelve years in the service of the hospital, a second addition of £5 a year is given, so that a private nurse then receives £55 per annum, and it is on a pension for this amount that she is entitled, if so disposed, to retire at the age of 45, after being 18 years a member of the London Hospital Nursing Staff. When any of our nurses are ill they not only receive free medical and nursing care, but practically all the expenses of illness are provided for, and they are given full pay during the whole illness. I have known this continued to a nurse for a year. These terms of pay and pension are a binding agreement with the committee. Although the official statement runs that such pension is paid "during the pleasure of the committee," this is merely a saving clause necessarily inserted because we should not continue a pension to a nurse who became, for example, a notorious criminal. At present we continue to pay half the premiums of 40 nurses who joined the Royal National Pension Fund for Nurses on the understanding that this help would be given to them. If nurses wish to join the Royal National Pension Fund independently, they are as much at liberty to do so as heretofore, and obviously this would not affect their claims to the promised hospital pension. At the other hospital, which you hold up as an example, there is a considerable and compulsory deduction from the nurse's pay each year. And now as to the general question. You write that everyone who pays a nurse has a right to expect that the nurse will receive practically the whole of what she may earn. This seems to me a strange argument. I cannot see what the person paying the nurse has to do with what becomes of the money. The nurse is a free woman at the London Hospital. Once she has served her four years, she has an absolutely free choice whether to go on with us or to leave and join the Co-operation or some similar nurse-providing institution, or, if she prefer it, to work on her own account. Considered only from a money point of view (and that is all I am discussing at the moment, as it is the point of the first part of your article) a nurse has to decide which is the best speculation—if you like so to put it—a certain £30 a year, rising £5 a year to £45, with an increase of £5 extra after six years' work and £5 more after 12 years, with washing and £4 for uniform, with free attendance and full pay during sickness, and full pay pension after 18 years' work—or the chance of earning, say, £80 to £100 in a lucky year, with all the attendant risks and expense of not being fully employed, the risks of illness and the certainty of no pension except such as she can secure for herself—say 10s. a week at 55 (10 years later than she would secure a much larger pension at the "London") if she had been able to put away £12 a year every year of her nursing work. It is not to be wondered at that the majority of our nurses prefer to remain, but even these are free to change their minds by giving a month's notice at any time. There are some people who delight to abuse the London Hospital system, but I feel sure that our system is successful because it is fair and advantageous to the nurses, and I know from daily and very carefully perused reports that we are successful in supplying to the public very good nurses indeed. I believe that their success is owing in great measure not only to their thorough training, but to the fact that their knowledge is kept up to date by their returning to the wards between their cases after sufficient rest. Medical men frequently attribute the efficiency of our private nurses to this amongst other causes, and I may mention, in conclusion, that only nurses holding the London Hospital certificate of training are appointed on our private staff.

[We do not think that Mr. Sydney Holland has made it quite clear in what way the £4,211 paid to the London Hospital for its private nursing institution fails to "presumably represent the profit made by the hospital from the earnings of the nurses"; for if the private nurses between their cases work in the wards it seems to us that the hospital owes to the home something more than their mere board and lodging; while if the hospital pays for the services thus rendered the amount paid cannot be set off against the £4,211 received from the home. With regard to our remarks on the question of pensions, we are confirmed in the view that there is no definite scheme which inalienably secures to the nurses a liberal pension after reaching a certain age, for according to the official statement which Mr. Holland quotes the pensions due are only payable "during the pleasure of the committee." A scheme founded on a proper business basis would assure a pension to every individual concerned who had lawfully earned one.—Ed. The Hospital.]

### Novelties for Nurses.

By Our Shopping Correspondent.

### A BANDAGE FOR DISTRICT NURSES.

Nurse Frances Dell has invented a chest bandage which claims to be "suitable for fixing dressings or poultices, and also acts as a good support." Nurse Dell, having worked in a country district for the past thirteen years, has noticed that it is very difficult for amateurs to fix bandages, and has therefore contrived this one which can easily be adjusted. The bandage, which is free from complications, is made of domette, and is therefore washable. It should be of great use to district nurses and amateurs for keeping poultices in place, and should constitute a good support. But I doubt whether, in many cases, it could be used advantageously for fixing dressings as it would be difficult to regulate the necessary pressure in certain places, and also would sometimes only afford insufficient protection. The bandage is called "The Dell," and is procurable from Messrs. W. H. Bailey and Son, Oxford Street, W. It might be usefully made in sizes small enough for the use of children.

### EUCINIA CREAM.

The address of the proprietors of this preparation, Messrs. Sandbrook and Co., is 448 Kingsland Road, N.E.

### The Acland Home at Oxford.

BY A CORRESPONDENT.

The combined work of the Sarah Acland Memorial and Acland Home has been carried on now for 25 years, and some review of its foundation and progress, with some account of its new constitution and rules, may be of interest. The first Sarah Acland nurse began work on January 1st, 1878, and the home has been so liberally supported by the general public, but more especially by the Oxford City Charity Trustees, that a superintendent, six district nurses, and two midwives are now employed.

### The Original and Subsequent Objects.

As stated in their first report, the immediate object of the original committee was—first, the gratuitous nursing of the sick poor in their own homes; second, provision for the care—on reduced terms of payment for the nurse—of persons whose circumstances were ascertained by previous inquiry to be such as to make them unable to meet the ordinary charges; third, to provide nurses for patients able to pay the usual prices. To this work was added in 1882 the reception as patients, primarily of undergraduates or of others who have not suitable accommodation in their own homes, or of those who come to Oxford from the country for medical and surgical treatment. The profits earned by the reception of patients, or by private nursing, helped to provide funds for more district nurses, and to assist such patients as were unable to pay the full fees.

### The Extension of the Movement.

In 1895 the work of the institution had extended so greatly that Northgate, a house in an excellent situation, and standing back in a large garden, was taken on a 99-years' lease from Lincoln College. Money collected as a testimonial to Sir Henry Acland, together with a sum of £1,000 contributed by Mr. Rudolf Lehmann in memory of H. B. Cotton, the well-known University oar, with donations generously given by many friends, and with the money realised by the sale of the houses in Wellington Square, was expended in building a wing for the reception of patients. It consists of a long corridor of two stories with patients' rooms on each floor, one of these being retained for the Rose Price patients. The bath-rooms and sanitary arrangements, all on the most approved modern type, are situated in a block towards the north, with cross-ventilation between them and the corridor. At present 14 patients can be admitted, and there is a staff of 20 nurses, some of whom attend private cases. A register of nurses resident in Oxford is also kept.

### A Royal Ceremony.

His Majesty the King, then the Prince of Wales, who opened the building in May 1897, said, in replying to the address made to him, "Nothing could have given me greater pleasure than to take part in this interesting ceremony, first of all because of my old Oxford days and the friends I made here, and for the sake of Mrs. Acland and her kind and excellent husband, Sir Henry. I sincerely wish this institution all possible success, and that it may grow bigger and bigger every year." For some time the district nurses lived in the old Northgate House, but it had long been felt that the institution had outgrown this arrangement, and in March 1903, Miss Denniston and the district nurses were moved temporarily into a house in Wellington Square.

### The New Regulations.

Until the present year the Committee of the Acland Home has consisted entirely of ladies, but under the new regulations gentlemen are also elected to serve on it. In November last a meeting of the subscribers was held in All Souls' College Hall under the presidency of Sir William Anson, and a strong special committee was appointed to consider the relation of the different branches of the work of the Acland Home, and to draw up a fresh constitution and rules. These were passed at a subscriber's meeting on February 27. They provide for an annual general meeting to be held in February at which patrons, vice-patrons, honorary secretary, honorary treasurer and committee are to be elected. The Committee shall present the report and accounts duly audited at the annual general meeting. Individual donors of £5 and upwards, persons who have been annual subscribers of two years of 10s. 6d. or more, and representatives of colleges, charities or other bodies contributing £5 or more, shall be entitled to vote at any general meeting. Special general meetings can be called by the Honorary Secretary on the written request of at least five subscribers. The Committee has power to appoint and dismiss the superintendents, salaried officers and nurses, to administer the funds of the Institution but not to incur a capital expenditure of more than £200 in any one year unless authorised to do so by a general meeting of the subscribers. They have also power to appoint three sub-committees to supervise the Sarah Acland Memorial District Nurses, the Acland Home, and the finances of the Institution, to which sub-committees all details of administration are delegated.

### A Benevolent Fund.

The regulations for the finance provide that out of the profits of the Acland Surgical and Medical Home provision shall be made each year for depreciation of lease, renewals, improvements, reserve and pensions, and unless the subscribers decide otherwise the surplus after paying interest at the rate of 5 per cent. on £1,534, being moneys specially given towards the District Nurses' Fund, and expended in the buildings of Northgate, shall be devoted to a Benevolent Fund for giving assistance to those who cannot afford the ordinary charges of a Nursing Home, or for providing nurses to private patients at reduced charges; assistance which has ever since the foundation of the Acland Home been largely given and very greatly appreciated.

### Progress of District Nursing.

For the year ending September 1882, the first year in which a list of cases attended and visits paid was published, the number of district cases nursed during the year was 170; total number of visits paid was 3,295; private cases nursed by the staff, 175; patients nursed in the Home, 7. During 1903 the figures were:—District cases nursed, 904; visits paid to district cases, 29,908; maternity cases, 132; nurses supplied to the poor for nights, 69. The Acland Medical and Surgical Home:—Private cases nursed by the staff, 212; patients nursed in the Home, 103. Of these last 19 patients were undergraduates and four ladies were admitted free under the Rose Price Benefaction. These figures show how the Institution has enlarged its sphere and now that the district nursing is definitely separated from the remunerative part it may be hoped that the Acland Home will, under its new constitution and rules, continue as his Majesty said in 1897 to grow bigger and bigger every year.

### Echoes from the Outside World.

### The King and Queen at Copenhagen.

On Good Friday, King Edward and Queen Alexandra, with Prince and Princess Charles of Denmark, attended Divine service at St. Alban's Church, Copenhagen, and in the afternoon the King, having called upon Sir W. E. Goschen at the British Legation, drove to the Ny Kongensgade to visit his old friend Count Mogens Frije, formerly Attaché at the Danish Legation in London, remaining for a couple of hours. On Saturday his Majesty received Dr. Deuntzer, the Premier and Minister for Foreign Affairs, but the audience was of an entirely private character. Both the King and Queen went to St. Alban's Church on Easter Day. On Monday the King received in audience at King Christian's Palace the members of the diplomatic body, and in the afternoon, his Majesty, accompanied by Prince John of Glücksburg, took a long walk in the city and environs, while the Queen, accompanied by her father, King Christian, went for a drive outside the capital. After dinner on Tuesday, the King of Denmark, the King and Queen of England, and the Royal Princes and Princesses, visited the Casino Theatre.

### Severe Fighting in Tibet.

There has been severe fighting in Tibet. The outbreak of hostilities which occurred last week at Hot Springs, Guru, was due to the Lhasan General himself, who, after inciting the Tibetans to throw stones, drew a pistol and shot a Sikh standing at his pony's head, smashing his jaw. This was regarded as a signal, and the Tibetans made a rush with their swords, after discharging all their firearms ready for use at the moment. The utter unexpectedness of the whole affair was most remarkable. The British casualties consisted only of a few wounded, a newspaper correspondent only being severely hurt. He was sitting writing when the onslaught was made and was unable to defend himself. The loss of the Tibetans exceeded 300 killed and many more wounded and prisoners. The killed included the Lhasan general and another general. It is clear that the Tibetans were determined to resist the advance of Colonel Younghusband's mission, as all the women and children had been sent away from Guru. On Sunday Colonel Younghusband visited the wounded Tibetans and told them that they would be allowed to return to their homes when they were cured. He added that the mission was still peaceful, and only wanted to treat with the Tibetans. The Tibet Mission, with the Flying Column, arrived at Guru on Monday afternoon. Colonel Younghusband was at once visited by a Chinese general from Lhasa, who, on behalf of the Amban, requested the mission to withdraw.

### The War in the Far East.

The Japanese House of Representatives signalised its final sittings by adopting a resolution to the effect that the whole nation was resolved to support the Emperor in the prosecution of a just war. In spite of the brilliant naval success, the final victory was still far off, but they would not hesitate to vote whatever expenses might be required. All tinned meats for the Russian army are being prepared by a German process, which enables the contents of each tin to be served hot without a fire. Port Arthur is gradually resuming its wonted animation. The place of the fugitives has now been taken in part by civilian officials of every description, who have been sent to fill the vacancies in the various public departments, and of course, to a much larger degree, by soldiers. Lately the weather has been splendid, and the entire population gathers twice a week to listen to the military band playing on the boulevard near the docks. The Japanese, continuing their advance northwards in Korea, have occupied Wiju, which their scouts found evacuated by the Russians. Everything is quiet at Niuchwang, where rapid progress is being made with the earthworks.

### The New High Commissioner in Cyprus.

The King has approved the appointment of Sir Charles Anthony King-Harman, Governor of Sierra Leone, to be his Majesty's High Commissioner in Cyprus, in succession to Sir William Frederick Haynes-Smith, whose term of office will shortly expire. The new Commissioner is 54, and started his official career, in 1874, as Private Secretary to the Governor of the Bahamas. He next became Private Secretary to the High Commissioner in Cyprus, and in the same year was appointed Assistant Commissioner of the Island. Subsequently, he was Assistant to the Chief Secretary of Cyprus, since which he has held several posts of importance in different colonies. His return to Cyprus as High Commissioner, after a lapse of 20 years, will be hailed with satisfaction.

### Death of a Judge.

On Monday evening Mr. Justice Byrne died at his residence, 33 Lancaster Gate, of acute bronchial pneumonia, supervening on a cold. The late Sir Edward Widdrington Byrne was a native of Islington, and was born 60 years ago. He was called to the Bar at Lincoln's Inn in 1867, took silk in 1888, sat in Parliament for five years, and was then elevated to the Bench in succession to Mr. Justice Chitty. He presided over the Chancery Division of the High Court, and was very popular in the profession, possessing also a large circle of private friends.

### Technical Education and Women Workers.

The London County Council have sent a circular letter to each of the Metropolitan Borough Councils stating that their Technical Education Board recently appointed a special sub-committee to inquire into the circumstances of women's work and the possibility of aiding the workers by means of technical instruction. The sub-committee, having made extensive inquiries on the subject, have come to the conclusion that very little can be done in the way of evening classes in trade subjects for women partly because of the apathy of working women as regards improvement in their trades, and partly because their long hours of labour leave them at the end of the day too tired to undertake further occupation of the same kind. The committee still, however, hope that something may be done by the co-operation of employers and of persons having apprenticing funds at their disposal by encouraging the attendance of girls and young women at technical institutes during working hours. For this reason the matter is being particularly brought under the notice of the Borough Councils.

### Easter Bank Holiday.

The returns of the railway companies show that a very large number of passengers left London for the Easter Bank Holiday. About 80,000 persons visited the Alexandra Palace, 78,000 the Crystal Palace, 39,000 Kew Gardens, and 16,000 the Tower of London. While there does not appear to have been a single accident on the railways, quite an alarming series of motor mishaps are reported. A collision near Hastings resulted in the injury of six persons; a little girl was run over and killed at Eastbourne; and on Sunday afternoon there was an upset at Grove Hill, near Harrow, which resulted in two deaths, while other members of the party in the motor-car were badly hurt. Several deaths from drowning took place on Monday, and a collision between a tramcar and a loaded brake at Portsmouth caused one death and severe injuries to four persons.

### A Book and its Story.

NEW NOVEL BY MR. EDEN PHILLPOTTS.*

Mr. Eden Phillpotts has chosen Dartmoor, and the war prison at Prince Town previous to the peace of 1815, for the background of his cleverly-constructed historical romance, "The American Prisoner." There is no lack of incident and movement in the book. Graphic pictures are given of the rough and ready manners of the time; mystery and tragedy hang round the loss of a valuable heirloom secreted in the cave of an old moor dweller—half fiend, half witch—formerly in the service of the Malherb family, from whom she had stolen treasure; and the sterner side of the story is relieved by the meeting of Grace Malherb and Cecil Stark, the young American officer rescued by her father, when nearly dead from exposure to a blizzard on Dartmoor, at the gates of his homestead, Fox Tor Farm. Stark was one of a small band of prisoners who had succeeded in making their escape from Prince Town. Their awful sufferings, after they found themselves outside the prison walls, face to face with the vast moor and the blinding storm, are told with painful reality.

"Now through the bursting heart of that great storm the American prisoners struggled on their way. None spoke, for all believed that death strode beside them and came closer with each savage thrust of the northern wind. About them the snow lay in a heavy carpet, and upon the moor, in gorges and deep ravines, an icy dust was piling into drifts that would only vanish with the suns of April. The gale blew with gigantic but irregular outbursts, so that it seemed as if fingers invisible on cruel hands stretched out of the night to tear their garments off them. The spirit of the storm escaped from its icy chambers, swept chill around them, and each breath they drew cut sharp into their lungs as the men panted onwards." Suddenly, through the mist they saw the gleam of a light, just at the moment when Cecil Stark's brother officer, Commodore Miller, was succumbing to the snow sleep. "Dear as the smile of a friend, as the sound of a voice, as the hand of a man stretched out to save, he had marked a ruddy flash from one little window high aloft on the western face of Fox Tor Farm. It flashed serene and steadfast; then the blizzard thundered down again and it vanished behind the snow."

Malherb, the owner of Fox Tor Farm, a man of restless nature and one whom "generosity and pride dominated in turn, whose failures were the work of other people, successes he claimed for himself," was also a victim of strong prejudices and an ungovernable temper. Hatred of the enemies of his country was one of his marked characteristics. On the night of the storm he was wandering restlessly round the homestead. His daughter, Grace, unable to sleep, lying awake and listening with strained ears to the storm without, heard the sound of a human voice through a lull in the tempest. "No other ear heard it, for though Malherb walked below, uneasy before the onset of this hurricane, his dwelling lay between him and the lost man; while for the rest, all that household slept in peace." Grace rose up and went out to her father, and together they heard the cry once more, "the last weak cry of the man in the snow as he called on the comrade who had perished beside him. "Miller! Miller! Mil—!" Then they heard no more, but, guided by the voice, struggled across the snow to it, and fell over a fellow-creature. Battered, bleeding, apparently lifeless, they discovered Cecil Stark, picked him up and thanked God, and carried him into Fox Tor Farm. For days the young officer lay unconscious of his surroundings; nor did his host realise whom he was harbouring. Stark had bought his clothes from a Jew pedlar, so he was not identified as a war prisoner by them. When he began to recover, he made himself known to Malherb. The announcement was received with astonishment and some indignation.

"Mr. Malherb, stamping into the parlour after his first conversation with the invalid, announced the discovery with considerable wrath. As yet no news of the outer world had reached Fox Tor Farm. It lay separated from all things by impenetrable barriers and drifts of snow. "An American! A wretched prisoner who broke out of Prince Town on the night of the storm. Doubtless that rascal who came so near to braining Grace in the summer. Thanks to Grace and to me he will regain his worthless life and not lose a finger." When Stark recovered sufficiently Grace was allowed to see him. He was at once recognised as the young American prisoner who some months previously had been brought to Grace's notice in a strange manner. Accompanied by Mr. Peter Norcott, a wealthy suitor whom she particularly disliked, Grace proposed to visit the church then being erected at Prince Town by the French and American prisoners. The Frenchmen chattered and sang to the clink of their trowels while within, thoughtful and more silent, a a hundred Americans were engaged upon carpentering and carving in wood and stone. Lieutenant Manwaring, the young officer in command of the works, took charge of the visitors, but he was called away for a moment, and before he departed he turned to an elderly American with grey hair and a distinguished bearing, and made a request. "May I ask you to show Miss Malherb and this gentleman round the works, Commodore Miller?" he said, and the prisoner bowed a grave assent. In looking at this man's sad eyes and noble face, Grace forgot the ridiculous rags that covered him. "Come this way, young lady," he said; "you see, our labours prosper. 'Twill be a monument for the generations that follow us. Our dust will mingle with this desert and be forgotten; our handiwork will remain." Mr. Peter Norcott's self-complacency received a shock as they proceeded; Grace remarked that the church was to be dedicated to St. Michael and All Angels. "Then," he responded, "you might have a personal interest in it; and may be I too shall have, for we might even be married here." "We might," was the girl's reply, "though not to one another." "Who knows? Time can work wonders." "But only God can work miracles," she rejoined. Suddenly a cry from overhead made Grace stop, start back, and look upward. It was a heavy chisel, which had dropped from a beam just missing Grace's head by a few inches. A cry rose on several lips: some shouted a curse at the man aloft on the beam from which the chisel had fallen; and Commodore Miller called out to him . . . but the man responsible for this accident was already half-way to the ground. He descended a rope ladder so quickly as to endanger his own neck. "I can only ask you to forgive me," he said; "I turned suddenly and my foot struck the chisel." "There's nothing to forgive," said Grace, "it was your voice which arrested me. If you had not shouted, I should not be here now; so I owe you nothing but gratitude." We cannot give the story away, for the romance that begins here, and the love affairs of Grace Malherb, with their tragic complications, are things to read about in the book itself. It is quite one of the best of its kind that has appeared this season.

* "The American Prisoner." By Eden Phillpotts. (Methuen. 6s.)

### Notes and Queries.

**FOR REGULATIONS SEE PAGE 277.**

*Home.*

(6) Can you tell me of a home where a feeble-minded man of the middle class could be taken care of? His friends could afford to pay about 12s. or 15s. weekly. I enclose stamped envelope, and trust that in asking for a private reply I am not transgressing the rules.—*M. G.*

Queries requiring private replies should be accompanied by 2s. 6d. Consult the Secretary, the National Association for Promoting the Welfare of the Feeble-minded, 58, Victoria Street, Westminster, S.W.

*Training of Male Nurses.*

(7) I am 22 years old, and have had three years' training in the Royal Army Medical Corps. Where can I apply for a Male Probationership?—*J.H.*

Probationers are received for 2 years' training at the National Hospital for the Paralysed and Epileptic, Queen Square, Bloomsbury. Salary, first year £12.

*Adoption.*

(8) Will you kindly tell me of a certificated nurse, having private means and a home of her own, who would adopt a child of good birth for a reasonable premium?—*Lonely.*

Perhaps the Association for Placing Orphans in Private Families might help you. Address the Hon. Secretary, 98, Cheyne Walk, London, S.W.

*Mental Nursing.*

(9) Having finished my general training, I should like to know if a certificate for mental nursing would be of much value to me; if so, where can I get information as to vacancies in asylums?—*Undecided.*

Certainly it would, if you take up work amongst the mentally afflicted. Vacancies are advertised in the Nursing papers.

*L.O.S.*

(10) Will you kindly tell me where I can obtain information about the London Obstetrical Society's examination?—*M.A.B.*

The Secretary, London Obstetrical Society, 20, Hanover Square, London, W.

*Hospital for Men.*

(11) Will you kindly tell me if there are any hospitals for men only, other than the Seamen's Hospital at Greenwich? Also if probationers are taken?—*C.B.*

Probationers are taken at the Seamen's Hospital, Greenwich. See the "Nursing Profession: How and Where to train" (Scientific Press) for further particulars.

*Post.*

(12) I am a trained nurse, 28, holding three-year certificate and excellent testimonials, both from Government and private appointments, and am desirous of obtaining a post as nurse-matron, or any position of responsibility. Have I any chance of obtaining such a position? and, if so, where and how shall I advertise?—*U.*

If you do not find what you want amongst the advertisements of appointments vacant, it would be well to advertise yourself in a Nursing journal with a large circulation. You appear well qualified, and, therefore, likely to succeed.

*Hospital Training.*

(13) A lady, over 40, wishes to give her services for 3 months only, in return for training in a London or suburban hospital. Can you tell to which hospitals to apply?—*Urgent.*

We cannot suggest an institution, as few require services such as you offer. Watch our advertisement columns or advertise yourself.

**Standard Nursing Manuals.**

"The Nursing Profession: How and Where to Train." 2s. net; 2s. 4d. post free.

"Nursing: Its Theory and Practice." By Percy Lewis, M.D. 3s. 6d. post free.

"Surgical Ward Work and Nursing." By Dr. A. Miles. 3s. 6d. net; 3s. 10d. post free.

"Elementary Physiology for Nurses." By C.F. Marshall, M.D., B.Sc., F.R.C.S. 2s. post free.

"Nurses' Dictionary of Medical Terms and Nursing Treatment." By Honnor Morten. 2s. post free.

"Fevers and Infectious Diseases." By W. Harding, M.D. 1s. post free.

"Art of Massage." By Mrs. Creighton Hale. 6s. post free.

### For Reading to the Sick.

"SOURCE OF MY LIFE'S REFRESHING SPRINGS."

Source of my life's refreshing springs,
Whose presence in my heart sustains me,
Thy love appoints me pleasant things,
Thy mercy orders all that pains me.

If loving hearts were never lonely,
If all they wish might always be,
Accepting what they look for only,
They might be glad—but not in Thee.

We need as much the cross we bear,
As air we breathe, as light we see,
It draws us to Thy side in prayer,
It binds us to our strength in Thee.

*A. L. Waring.*

In special undertakings and missions God makes use of us as instruments. His providence works by means of us as His instruments, and undoubtedly it enters within the scope even of our personal trial, acting well as such instruments. Still, if we view those capacities in which we act a part, it must be seen that what we do as instruments is a far inferior work to that which we do to fulfil our own personal trial. . . . We are not united to God simply by being used by Him as means: this is an office which belongs to good and bad alike. But in fulfilling the purpose of life as a sphere of trial and probation, we are God's servants in a different sense from that in which His instruments are; we are His servants by personal obedience, and by the agreement of our wills to His will. . . . The divine object may be answered, and the instrument who is used to accomplish it may be condemned, condemned even for the very spirit which he has shown while acting as such an instrument. The general end of life, as trial, is superior to all special ends, and it is thus superior because the general end is also the individual end. It is the end which concerns the individual being, his spiritual condition, his ultimate prospects. It contains the highest and grandest ends to him, the only ends that are of any moment to him; it contains an eternal end—the reward of life everlasting.—*J. B. Mozley, D.D.*

Each of us has some intended niche to occupy, and some one particular work to do, just as surely as it was the work of our Divine Redeemer, and of Him alone, to achieve the salvation of the world. It may be a task of many years. It may be a single action, a single witness to truth, a single act of duty, done at one particular day, at one hour—nay in the compass of a few minutes, yet carrying in it all the moral power of a lifetime—and, exhausting by being done, the reasons for which in the Eternal Mind life was given to the agent.—*Dr. Liddon.*

There is only one thing in this world greater than happiness, and that is holiness; and it is not in our keeping; but what God has put in our power is the happiness of those about us, and that is largely secured by our being kind to them.—*Anon.*

Be with God in thy outward works, refer them all to Him. Seek to do them in Him and for Him and He will be with thee in them, and they shall not hinder but rather invite His presence in thy soul. Seek to see Him in all things, and in all things He will come nigh to thee.—*E. B. P.*

